# Pathways and disease-causing alterations in visual chromophore production for vertebrate vision

**DOI:** 10.1074/jbc.REV120.014405

**Published:** 2020-11-23

**Authors:** Philip D. Kiser, Krzysztof Palczewski

**Affiliations:** 1 The Department of Physiology & Biophysics, University of California, Irvine, California, USA; 2 Research Service, The VA Long Beach Health Care System, Long Beach, California, USA; 3 The Gavin Herbert Eye Institute, Department of Ophthalmology, University of California, Irvine, California, USA; 4 The Department of Chemistry, University of California, Irvine, California, USA

**Keywords:** rhodopsin, visual cycle, retinoid cycle, RPE65, RGR, retina, eye, G protein-coupled receptor (GPCR), 11cRAL, 11-cis-retinal, 11cRDH, 11-cis-RDH (RDH5), 11cRE, 11-cis-retinyl esters, 11cROL, 11-cis-retinol, AAV, adeno-associated virus, ABCA4, ATP-binding cassette subfamily A member 4, adRP, autosomal dominant retinitis pigmentosa, AMD, age-related macular degeneration, arRP, autosomal recessive RP, atRAL, all-trans-retinal, atRDH, all-trans-RDH, atRE, all-trans-retinyl esters, atROL, all-trans-retinol, AWAT2 (MFAT), acyl-CoA wax alcohol acyltransferase 2, BCO, β-carotene 15,15′-dioxygenase, CCD, carotenoid cleavage dioxygenase, CMV, cytomegalovirus, CRALBP, retinaldehyde-binding protein, CRISPR/Cas9, clustered regularly interspaced short palindromic repeats and CRISPR-associated protein 9, CSNB, congenital stationary blindness, DSBs, DNA double-stranded breaks, ER, endoplasmic reticulum, FDA, U.S. Food and Drug Administration, GLF, gain and loss of function, GoF, gain of function, HDR, homology-directed repair, HRAS, HRas proto-oncogene (GTPase), IRBP, interphotoreceptor retinoid-binding protein, LCA, Leber congenital amaurosis, LoF, loss of function, LRAT, lecithin:retinol acyltransferase, Luxturna, voretigene neparvovec-rzyl, ND, nitrosotalea devanaterra, NIH, National Institutes of Health, NHEJ, nonhomologous end joining, NinaB, neither inactivation nor afterpotential mutant B carotenoid oxygenases, NRL, retina-specific leucine zipper protein, OS, the photoreceptor outer segments, PAM sequence, protospacer adjacent motif, PDB, Protein Data Bank, RCD, rod–cone dystrophy, RDH, retinol dehydrogenase, RGR, retinal G-protein-coupled receptor, Rho, rhodopsin, RNAseq, next-generation RNA sequencing, RP, retinitis pigmentosa, RPE, retinal pigment epithelium, RPE65, retinoid isomerase

## Abstract

All that we view of the world begins with an ultrafast *cis* to *trans* photoisomerization of the retinylidene chromophore associated with the visual pigments of rod and cone photoreceptors. The continual responsiveness of these photoreceptors is then sustained by regeneration processes that convert the *trans*-retinoid back to an 11-*cis* configuration. Recent biochemical and electrophysiological analyses of the retinal G-protein-coupled receptor (RGR) suggest that it could sustain the responsiveness of photoreceptor cells, particularly cones, even under bright light conditions. Thus, two mechanisms have evolved to accomplish the reisomerization: one involving the well-studied retinoid isomerase (RPE65) and a second photoisomerase reaction mediated by the RGR. Impairments to the pathways that transform all-*trans*-retinal back to 11-*cis*-retinal are associated with mild to severe forms of retinal dystrophy. Moreover, with age there also is a decline in the rate of chromophore regeneration. Both pharmacological and genetic approaches are being used to bypass visual cycle defects and consequently mitigate blinding diseases. Rapid progress in the use of genome editing also is paving the way for the treatment of disparate retinal diseases. In this review, we provide an update on visual cycle biochemistry and then discuss visual-cycle-related diseases and emerging therapeutics for these disorders. There is hope that these advances will be helpful in treating more complex diseases of the eye, including age-related macular degeneration (AMD).

Visual sensation is a complex process whereby light from the environment is transduced into a bioelectrical signal that is preprocessed by the retina into a form suitable for further interpretation by the brain. The light-detection step of this pathway utilizes a set of G-protein-coupled retinylidene opsin proteins known as visual pigments that are located in the photoreceptive neurons of the retina. Visual pigments contain a covalently bound 11-*cis*-retinal chromophore that undergoes *cis*–*trans* isomerization upon absorption of visible light (1) (Movie S1). This photoswitch enables the visual opsin to trigger a G protein signaling pathway that ultimately leads to photoreceptor cell hyperpolarization and a change in chemical signaling at the first visual synapses between photoreceptors and bipolar cells (2, 3, 4). Unlike most other retinylidene proteins in various kingdoms, the vertebrate visual pigments do not retain their retinal chromophore following photoisomerization (5). Rather, the Schiff base linkage becomes susceptible to hydrolysis in the activated state of the receptor, leaving the visual opsin insensitive to further light stimulation. The spent chromophore must then be regenerated and combined with opsin to enable another round of light detection. A key innovation that evolved in vertebrates is the ability to convert all-*trans*-retinal into 11-*cis*-retinal in the absence of light. The metabolic pathway responsible for this activity, which is known as the classical visual or retinoid cycle, was discovered over 150 years ago in frog (translated in Ref. (6)) and is now understood in great molecular detail (7, 8). By contrast, only lately have light-dependent mechanisms (collectively referred to here as nonclassical visual cycles) of 11-*cis*-retinal biosynthesis become more generally recognized as important in maintaining vertebrate visual pigment sensitivity under conditions of high illuminance.

Because the visual system depends so critically on these 11-*cis*-retinal renewal pathways, it is not surprising that mutations in the key proteins that mediate the all-*trans* to 11-*cis*-retinal conversion lead to visual defects. In some cases, visual cycle protein mutations produce blindness within the first or second decade of life (9), which has motivated studies to develop therapeutics for such conditions. These include pharmacological agents that bypass the metabolic blockade in 11-*cis*-retinal synthesis (10, 11) and targeted genetic approaches involving gene augmentation or mutation correction using CRISPR/Cas9 or related technologies (12). Because the retina is well isolated in the ocular globe from the remainder of the body, gene therapies can be delivered to the intended site of action with reduced concern for off-target systemic side effects. Hence, ocular gene therapy is one of the most advanced branches of this field and has reached key milestones for the discipline, including the first U.S. Food and Drug Administration (FDA)-approved gene therapy for an inherited disease (13).

Here, we first review recent progress in understanding how 11-*cis*-retinal is produced through both the classical and nonclassical visual cycles. Recent reevaluation of the potential role of the retinal RGR in chromophore production adds to the complexity of this key visual process. Next, we follow with a discussion of how specific mutations in visual-cycle-related proteins lead to retinal disease and blindness. Finally, we review progress made in the treatment of such disorders with a focus on pharmacological and genetic approaches. Where appropriate we cite other more specialized review articles, which deal with unique aspects of the visual cycle.

## Expression of visual cycle proteins in the vertebrate retina

**Figure 1:**
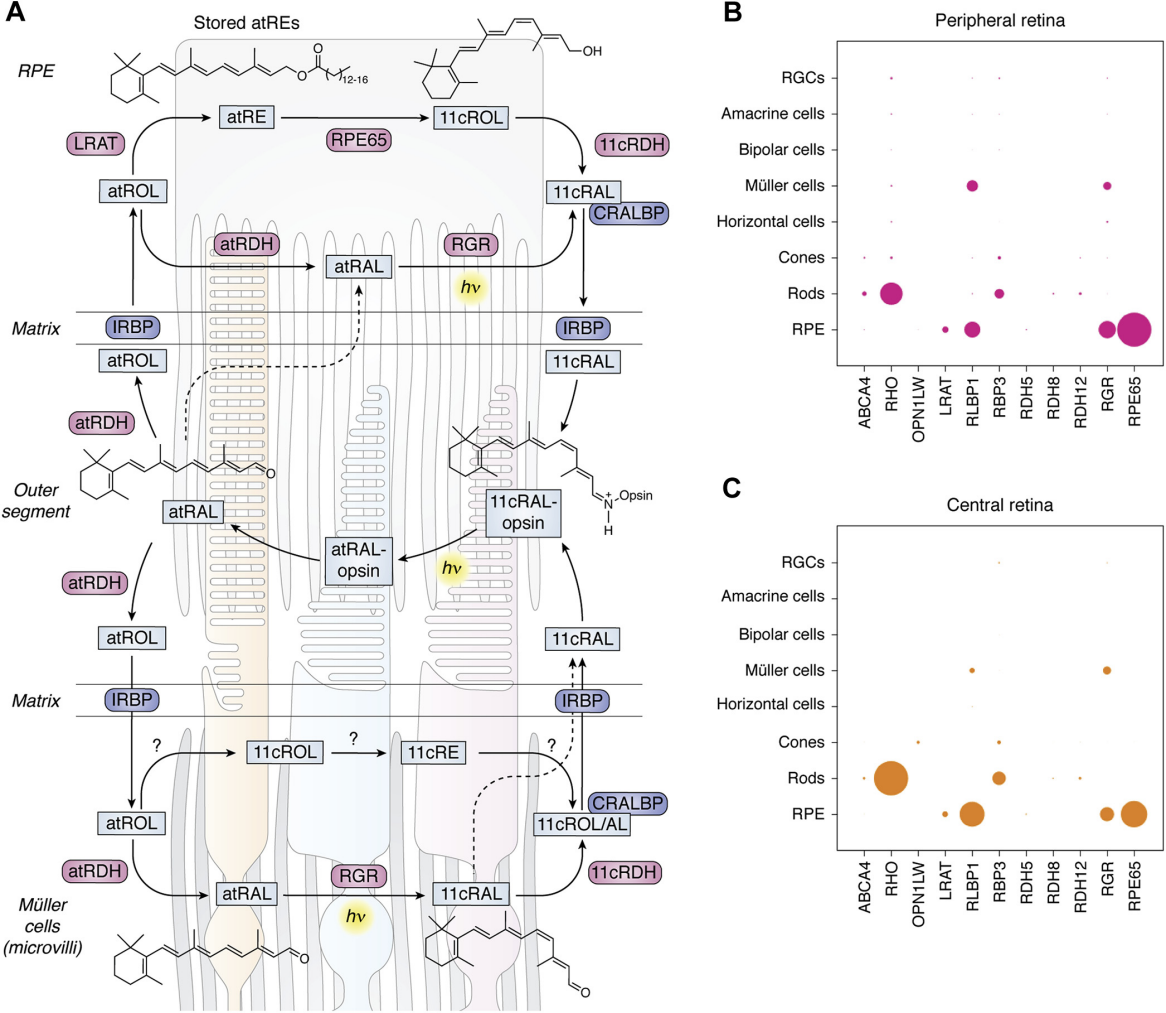
**Localization and function of proteins involved in visual chromophore production in the vertebrate retina.***A*, vision is initiated by the absorption of light (hν, *yellow highlight*) by visual pigments localized in the photoreceptor outer segments (central region of the panel; rod outer segment on the left, cone outer segments in the middle and right). These outer segments are connected to the photoreceptor inner segments and cell bodies shown in the bottom portion of the panel (unlabeled). Protein-mediated pathways required for 11-*cis*-retinal synthesis and the regeneration of the visual pigment molecules reside in the RPE (upper panel) and Müller cells [bottom panel; note: only the Müller cell microvilli (wavy gray structures) are shown]. Enzymes and retinoid-binding proteins are shown in pink and dark blue boxes, respectively. Note the presence of light-dependent and light-independent pathways in both RPE and Müller cells. *Dashed lines* indicate retinoid diffusion/transport pathways of unclear function. Abbreviations are as follows: 11cRAL, 11-*cis*-retinal; 11cRDH, 11-*cis*-RDH; 11cRE, 11-*cis*-retinyl esters; 11cROL, 11-*cis*-retinol; atRAL, all-*trans*-retinal; atRDH, all-*trans*-RDH; atRE, all-*trans*-retinyl esters; atROL, all-*trans*-retinol; CRALBP, cellular retinaldehyde-binding protein; IRBP, interphotoreceptor retinoid-binding protein; RGR, retinal G-protein-coupled receptor; RDH, retinol dehydrogenase. Question marks indicate steps where a physiologically relevant enzyme has yet to be identified. *B*–*C*, single-cell RNA sequencing analysis of visual-cycle-associated proteins in the peripheral (*B*) and central (*C*) regions of the human retina. The area of each circle indicates the expression level of the given gene (shown on the horizontal axis) in each cell type (vertical axis). Data are taken from reference (18).

Generation of the visual chromophore in the vertebrate retina involves 4 cell types: rod and cone photoreceptor neurons, the retinal pigment epithelium (RPE), and the Müller glia (Fig. 1*A*). Originally, it was believed that the RPE’s principle role was the production of visual chromophore for visual pigments in rod photoreceptor cells, whereas the Müller glia primarily supported cone pigment regeneration. Newer data have blurred these distinctions, and an interdependence of the classical and nonclassical visual cycles is becoming increasingly clear (14, 15, 16).

The cellular localization of proteins comprising the visual cycle was originally deduced primarily from traditional protein purification approaches and antibody-based methods such as immunohistochemistry. These studies led to the picture of visual cycle protein expression shown in Figure 1*A*, with the protein machinery necessary to form 11-*cis*-retinal distributed between various cell types. More recently, the advent of high-throughput RNA sequencing technology has allowed regional expression levels and the degree of expression variability to be assessed at the single-cell level (Fig. 1, *B*–*C*) (17, 18). These single-cell RNAseq (next-generation RNA sequencing) results agree well with the original localization assignments based on expression at the protein level and have revealed additional finer details in visual cycle protein expression, for example, between the cone-rich central region of the human retina and the rod-rich periphery.

Visual cycle enzymes specifically expressed within the RPE include lecithin:retinol acyltransferase (LRAT), RPE-specific 65 kDa protein (RPE65 retinoid isomerase), and an 11-*cis*-retinol dehydrogenase (11cRDH) known as RDH5. As shown in Figure 1*A*, these enzymes sequentially produce all-*trans*-retinyl esters, 11-*cis*-retinol, and 11-*cis*-retinal from all-*trans*-retinol that is formed within rod and cone OSs. RPE65 was also detected within cone photoreceptors and proposed to play a role in cone pigment regeneration (19); however, this was not confirmed by RNAseq analysis (Fig. 1, *B*–*C*) or by direct functional assessment (20).

Other proteins such as cellular retinaldehyde-binding protein (CRALBP) and RGR are expressed in both Müller glia and the RPE and may play distinct functional roles in these two cell types. CRALBP is a member of the CRAL-TRIO superfamily that binds 11-*cis*-retinal and 11-*cis*-retinol under physiological conditions (21, 22). In the RPE, CRALBP binds 11-*cis*-retinal, which it obtains from 11-*cis*-RDHs (*e.g.*, RDH5) as well as RGR. In the Müller glia of bovine retina, CRALBP is associated with both 11-*cis*-retinal and 11-*cis*-retinol. This finding is notable given that 11-*cis*-retinol is readily oxidized to 11-*cis*-retinal within the OSs of cones but not those of rods (23, 24). Hence, CRALBP within Müller glia plays a key role in supplying cones with a form of visual chromophore that cannot be used by rods. RNAseq analysis showed that CRALBP expression is lower in the cone-rich central retina of humans in contrast with the retinal periphery. These differences possibly reflect the reduced need of cones for a privileged chromophore supply when the density of rods is low.

Another retinoid-binding protein, interphotoreceptor retinoid-binding protein (IRBP), functions as a retinoid carrier (buffer) within the interphotoreceptor matrix that separates the RPE and the photoreceptors (Fig. 1*A*). The protein is synthesized by photoreceptors and is secreted into the interphotoreceptor matrix in a highly glycosylated form. IRBP binds all-*trans* and 11-*cis*-retinoids in addition to unsaturated fatty acids within its four homologous modules (25, 26, 27). Using *Irbp*^-/-^ mice it was shown that this protein is not essential for visual cycle operation (28, 29). Nevertheless, these mice exhibit time-dependent rod and cone degeneration (30). Further studies have shown that IRBP prevents formation of potentially toxic lipofuscin through its retinoid-binding action (31).

The structure of IRBP comprises four homologous modules. Crystal structures of two modules were reported (32, 33), revealing a conserved two-domain architecture consisting of an N-terminal ββα-spiral fold linked to a C-terminal αβα sandwich. The structure of full-length bovine IRBP was solved at a global resolution of 8.1 Å using cryoelectron microscopy (27), revealing a bent filament or “π”-shaped structure 14.1 nm in diameter, 39.1 nm in length, and 2.2 to 4 nm in width. This structure would enable IRBP to wrap around an unknown anchor to be retained in the extracellular space between the photoreceptors and RPE to prevent rapid clearance by the RPE.

RGR, a seven-transmembrane spanning protein that appears to function as a retinal photoisomerase, not as the classical GPCR suggested by its name, is also expressed in both the RPE and Müller glia and may have distinct functional roles in the two cell types. In the RPE, RGR has been proposed to support visual chromophore production by a variety of mechanisms including acting directly as a photoisomerase (34, 35, 36), by stimulating the isomerase activity of the classical visual cycle (37, 38), or by clearing potentially toxic isomers of retinal (39). RGR may form 11-*cis*-retinal through its photoisomerase activity in conjunction with an 11-*cis*-RDH (40), (*e.g.*, RDH10 (35)), although the *in vivo* role of this enzyme has not been validated (41). Regardless of the exact mechanisms of RGR, the impaired cone function observed in *Rgr*^-/-^ mice clearly shows that this protein plays an important role in visual chromophore production (35). However, RNAseq analysis has demonstrated that RGR expression is much lower in mouse Müller glia as compared with human and bovine Müller glia indicating that the phenotype in *Rgr*^-/-^ mice may not fully recapitulate the consequences of RGR loss of function (LoF) in other species (36). As shown in Figure 1*A*, the full complement of proteins that contribute to visual chromophore production in the Müller glia remains to be identified, and some previously suspected players such as Des1 have been shown physiologically irrelevant to this process (15, 42). Others such as acyl-CoA wax alcohol acyltransferase 2 (AWAT2 or MFAT), which catalyzes stereospecific esterification of 11-*cis*-retinol, require further study (43, 44).

## Evolution of the 11-*cis*-retinal synthetic machinery in vertebrates

**Figure 2:**
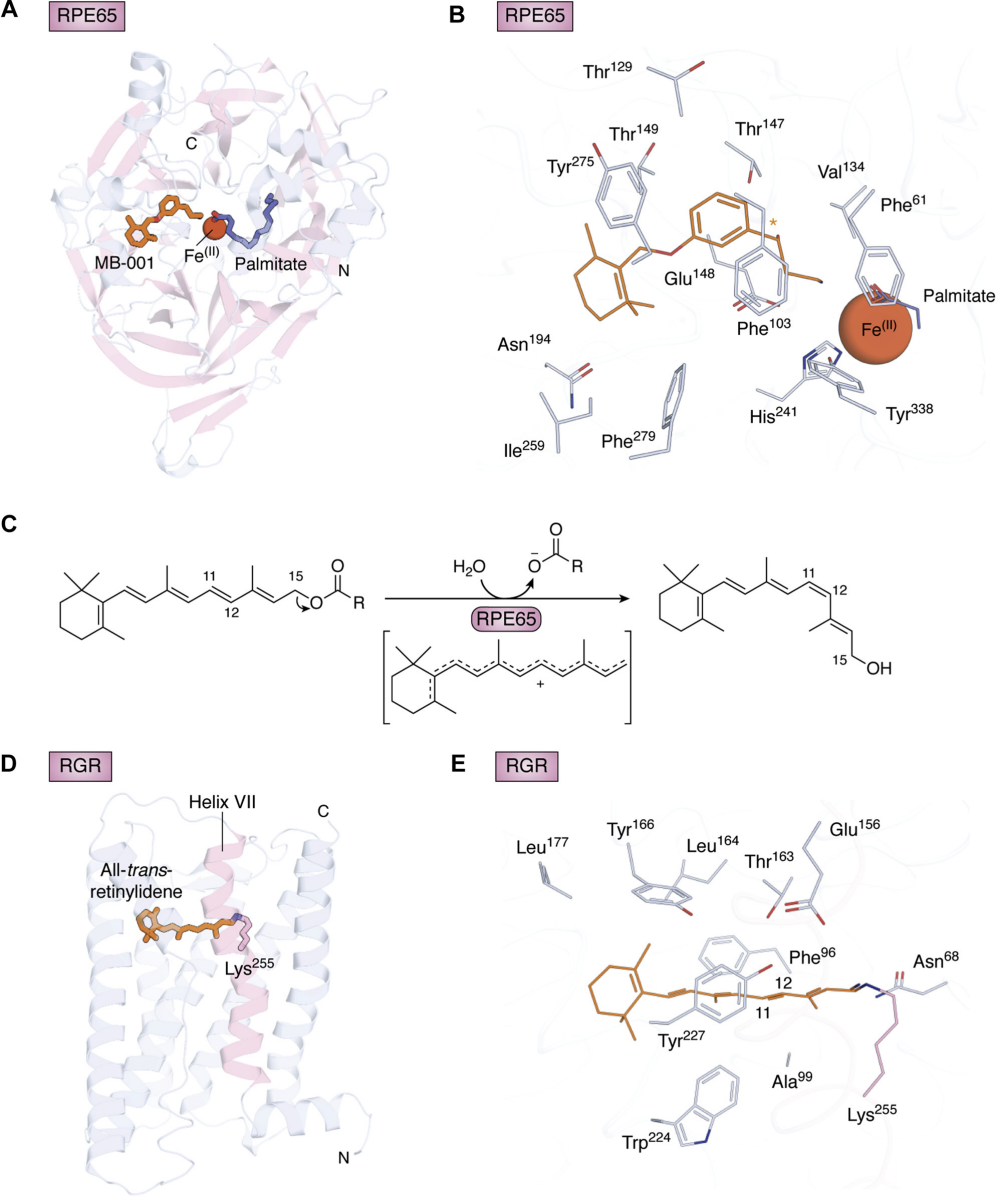
**Three-dimensional structural models of visual cycle retinoid isomerases.***A*, crystal structure of bovine RPE65 in complex with MB-001 (*orange sticks*), an 11-*cis*-retinoid mimetic, and the palmitate product of catalysis (*purple sticks*) (Protein Data Bank (PDB) accession code 4RSE). *B*, detailed view of the RPE65 active site showing residues in proximity to the retinoid-binding site. The orange asterisk indicates the predicted binding position of the retinoid C^11^-C^12^ bond. The iron ion is directly coordinated by a set of 4-His residues (only His^241^ is shown in the figure). Thr^147^, Phe^103^, Tyr^338^ play key roles in polyene isomerization stereoselectivity (54, 81, 82). *C*, RPE65-catalyzed isomerization reaction involving a putative carbocation intermediate. *D*, homology model of human RGR bound to all-*trans*-retinal *via* a Lys^255^ Schiff base. The model was generated using bovine rod opsin as a template (PDB accession code 3PXO) *via* the SWISS-MODEL server (183). Helix VII containing the chromophore-binding Lys^255^ residue is shown in pink. *E*, predicted model of the RGR active site. Residues that are absolutely conserved across a wide range of species from humans to zebrafish and within 4.5 Å of the retinylidene group (*orange lines*) are shown as lines. Aromatic residues Tyr^166^, Phe^96^, Tyr^227^, Trp^224^ could be critical in *trans*–*cis* stereospecificity, and Glu^156^ could be a counterion of the protonated Schiff base between retinal and Lys^255^.

Like many other evolutionary processes, the advent of the vertebrate visual cycles involved the repurposing of several existing protein scaffolds rather than *de novo* invention of novel enzymes and retinoid chaperones. Components of the classical visual cycle are believed to have come into existence approximately half a billion years ago as the vertebrate lineage emerged from the last common ancestor of chordates (45, 46, 47). The visual cycle as well as visual opsin evolution is thought to have been driven by a change in visual ecology as vertebrate predecessors began to occupy deep waters where light-driven 11-*cis*-retinal production became a nonviable pathway (48). The visual opsins evolved to become more photosensitive and achieve greater efficiency in activating G proteins while at the same time losing their ability to remain covalently linked in the binding pocket in *cis* and *trans* configurations (49, 50). Support for this timeline comes from biochemical and phylogenic studies on the presence of RPE65 and LRAT activities, the two essential enzymes of the classical visual cycle, in urochordates, cephalochordates, and in the early branching cyclostome vertebrate lineage represented by sea lampreys (51). RPE65, a retinyl ester isomerase, belongs to the carotenoid cleavage dioxygenase (CCD) superfamily whose members typically cleave alkene bonds in carotenoid substrates (52). LRAT belongs to the HRas proto-oncogene (HRAS)-like tumor suppressor family, whose members in turn are circularly permuted variants of papain-like thiol peptidases of the NlpC/P60 superfamily (53). Despite having undergone dramatic changes in their catalytic activities, these enzymes retain use of key active site elements present throughout their respective families—namely a 4-His-coordinated Fe^II^ cofactor in the case of RPE65 (54, 55) and a Cys-His-His catalytic triad in the case of LRAT (56, 57) (Fig. 2, *A*–*B*). Although an RPE65 ortholog (BCMOa) was previously reported in the tunicate *Ciona intestinalis* based on amino acid sequence similarity (58), it was later shown through biochemical assays that this enzyme instead possesses carotenoid oxygenase activity (51, 59). Conversely, the RPE65 ortholog from sea lamprey has demonstrable retinoid isomerase activity (51), which supports the idea that RPE65 appeared in the last common ancestor of gnathostomes and cyclostomes following an extensive divergence from a β-carotene 15,15'-dioxygenase (BCO)-like ancestor (59). Likewise, sea lamprey LRAT is a functional retinyl ester synthase (51), and phylogenic analyses indicate that true LRAT orthologs are also restricted to vertebrates (46). Similar observations have been made for vertebrate retinol dehydrogenase 5 (RDH5) and RDH8, both of which belong to the short-chain dehydrogenases/reductases (SDR) family and share most recent common ancestors with hydroxysteroid dehydrogenases (46, 60). The fact that vertebrate visual cycle RDHs do not form a monophyletic group indicates either that the ancestral enzyme was promiscuous with the ability to metabolize retinoids, steroids, and perhaps other lipophilic molecules or that RDH activity has been acquired multiple times through convergent evolution. RGR, on the other hand, is orthologous to opsins found in invertebrates, the best characterized of which is a protein known as retinochrome (61, 62, 63). Similarly, distant CRALBP orthologs are found in mollusks and other invertebrates (46, 64), where they play a role in supporting RGR photoisomerase activity by acting as retinal shuttles (65). CRALBP appears to play a similar role in vertebrates by stereospecifically binding 11-*cis*-retinal and 11-*cis*-retinol. The RGR-photoisomerase system clearly originated before the classical visual cycle, likely to support the generation or regeneration of opsins under conditions where the organism was frequently exposed to a well-lit environment. Such activity may be retained in vertebrates today in the regeneration of cone visual pigments, which are operative under conditions where photon flux onto the retina is high.

## Mechanisms of retinoid isomerization

All-*trans* to 11-*cis*-retinoid isomerization is the hallmark step of the recycling portion of the visual cycle and is a process highly specific to the eye, as 11-*cis*-retinoids are found in significant quantity only in the retina. Both enzymatic and photoenzymatic retinoid isomerization occurs within the membranes of the endoplasmic reticulum (ER) where the isomerases interact with other components of the visual cycle enzymatic machinery (66, 67, 68). As described in detail previously (69), the isomerization reaction requires energy, because 11-*cis* retinoids are higher in free energy than all-*trans*-retinoids primarily due to steric factors (70, 71). Additionally, the isomerization mechanism must involve lowering the bond order of the C^11^-C^12^ double bond to allow for the molecular rotation, which ordinarily would be kinetically unfavorable due to the rigidity of the polyene backbone (72).

Before retinoids are isomerized by RPE65, they must be esterifed by LRAT (73, 74, 75, 76). The enzyme first forms a thioester catalytic intermediate, and subsequently the acyl group is transfer on all-*trans*-retinol (56). The mechanism of this reaction is reviewed in Ref. (77).

The mechanism of RPE65-catalyzed isomerization has been elucidated over the course of several years through the work of many independent groups. Although certain aspects of RPE65 action require additional clarification, there is now a reasonable consensus in the field concerning the details of its catalytic nature (52, 55, 78). In contrast to alkene-cleaving CCDs, which use their iron prosthetic group to trigger O_2_ reactivity (79), RPE65 appears not to require O_2_ as a cosubstrate or cocatalyst to carry out retinoid isomerization (80). Structural studies have suggested that iron instead serves as a Lewis acid catalyst to facilitate ester bond hydrolysis, which likely pays for the thermodynamic debt incurred from the *trans*–*cis* isomerization (81) (Fig. 2*A*). It is notable that ester cleavage occurs at the C^15^–O position, as demonstrated by numerous isotope labeling studies ((82, 83) reviewed in (67)), rather than at the acyl bond more typical of biological ester hydrolysis. As shown in Figure 2*C*, *O*-alkyl cleavage is favorable because of the proximity of C^15^ to the conjugated polyene, which stabilizes the cationic intermediate. Crucially, electron withdrawal through *O*-alkyl cleavage appears to be mechanistically essential as the retinyl cation intermediate exhibits lowered bond order required for efficient geometric isomerization (72). Through analysis of the RPE65 active site in the presence of putative transition state analogs, it was proposed that the retinyl cation is preferentially stabilized at C^11^ through electrostatic interactions involving the side chains of conserved residues Phe^103^ and Thr^147^ (81) (Fig. 2*B* and Movie S2), as previously indicated through detailed mutagenesis experiments (47, 83). Structural comparison of RPE65 to an archaeal carotenoid-cleaving CCD from *Nitrosotalea devanaterra* (*Nd*CCD) with a metazoan CCD-like active site revealed a key difference in active site shape between these two proteins that likely plays a decisive role in determining whether or not the polyene is isomerized upon carbocation formation (84). In RPE65, the retinoid-binding site has a curved shape that likely helps force the retinoid substrate from a straight *trans* geometry into the bent 11-*cis*-configuration of the retinol product. The apocarotenoid-binding site of *Nd*CCD overlaps with the retinoid-binding site of RPE65 but exhibits a linear geometry, consistent with the finding that *Nd*CCD does not isomerize its substrates during oxygenolysis of the target alkene bond despite the fact that a polyene cationic intermediate is believed to be formed during its catalytic cycle (79). Importantly, the possibility of concomitant alkene cleavage and alkene isomerization was demonstrated through the discovery of NinaB (neither inactivation nor afterpotential mutant B carotenoid oxygenases that possess such isomerase activity combined with oxygenase activity as part of a putative insect visual cycle (85). Further comparative analysis of alkene-cleaving CCDs with RPE65 is likely to provide deep insights into common and divergent catalytic properties within this enzyme superfamily.

## Photoisomerization and RGR

In contrast to the “dark” *trans*–*cis* isomerization carried out by RPE65, RGR opsin employs the energy of a photon to overcome both kinetic and thermodynamic barriers to 11-*cis*-retinal formation from all-*trans*-retinal substrate. As in other retinylidene proteins, photon absorption generates an excited state of the chromophore with an electron density distribution that is conducive to C^11^-C^12^ bond rotation (reviewed in (5)). Additionally, the energy carried by a 500 nm photon (∼57 kcal/mol) is more than needed to pay for the endergonic transition from an all-*trans* to an 11-*cis* configuration. While the specifics of RGR-opsin activity remain to be elucidated, the general principles by which this photoenzyme functions can be deduced by comparison to other well-characterized retinylidene proteins. In addition to all-*trans*-retinal, RGR was shown to bind13-*cis*-retinol, which is the undesirable by-product of RPE65 catalysis and nonspecific thermal isomerization. Upon absorption of visible light by the retinylidene adduct, RGR isomerizes both all-*trans*-retinal and 13-*cis*-retinol to the 11-*cis*-configuration (36, 86). Unlike most other nonvisual opsin proteins, which retain their retinal ligand in both the *cis* and *trans* forms and can be interconverted through stimulation by distinct electronic absorbance bands, the 11-*cis*-retinylidene complex with RGR is unstable and susceptible to hydrolysis, particularly in the presence of the 11-*cis*-retinal acceptor, CRALBP (36) or potentially RDH enzymes (35). The all-*trans*-retinal–RGR complex exhibits two main absorbance maxima at 470 and 375 nm, which represent the protonated and deprotonated forms of the retinylidene Schiff-base linkage (87). There are conflicting data regarding the relationships between these absorbance bands and the action spectrum of RGR. The initial characterization of RGR opsin (86) as well as a more recent analysis (35) has suggested that the action spectrum overlaps with the 470 nm absorbance band. In a separate report, it was demonstrated that both RGR from bovine RPE microsomes and RGR produced heterologously in HEK293 cells exhibit maximal activity when exposed to 505 to 535 nm light (36). The origin of these differing results requires further research to resolve but may involve variable levels of competing back-photoisomerization processes as observed previously for the photoisomerase opsin retinochrome (88). A high-resolution structure of RGR opsin has not yet been determined, and existing opsin structures have low sequence identity to RGR opsin (∼25%). Although homology modeling is expected to have limited accuracy under these circumstances, Figure 2, *D*–*E* display a preliminary view of the RGR-opsin structure based on the crystal structure of bovine rod opsin bound to all-*trans*-retinal (89). In this model RGR opsin adopts the seven-transmembrane structure characteristic of retinylidene proteins with the N-terminus facing the ER lumen and the C-terminus facing the cytosol. Retinal is bound in a pocket closer to the ER lumen *via* a Schiff-base linkage involving Lys^255^, which is homologous to Lys^296^ in bovine rod opsin. Figure 2*E* shows residues, conserved among vertebrates, predicted to line the retinal-binding pocket. Notable potential interactions include Glu^156^ serving as a potential counterion to the protonated Schiff base; and Trp^224^ and Thr^163^ forming a possible aromatic–dipole interaction at the site of polyene isomerization, like the interaction observed in the RPE65 active site. The Phe side chain of position 96, a character state specific to the RGR-opsin lineage, forms a close interaction with the retinal C^19^ methyl group, suggesting that it may play an important steric role in the photoisomerization reaction. Continued structure/function studies of RGR opsin will be critical for resolving its detailed mechanism of catalysis.

## Genetic disease-causing changes in the visual cycle

**Figure 3:**
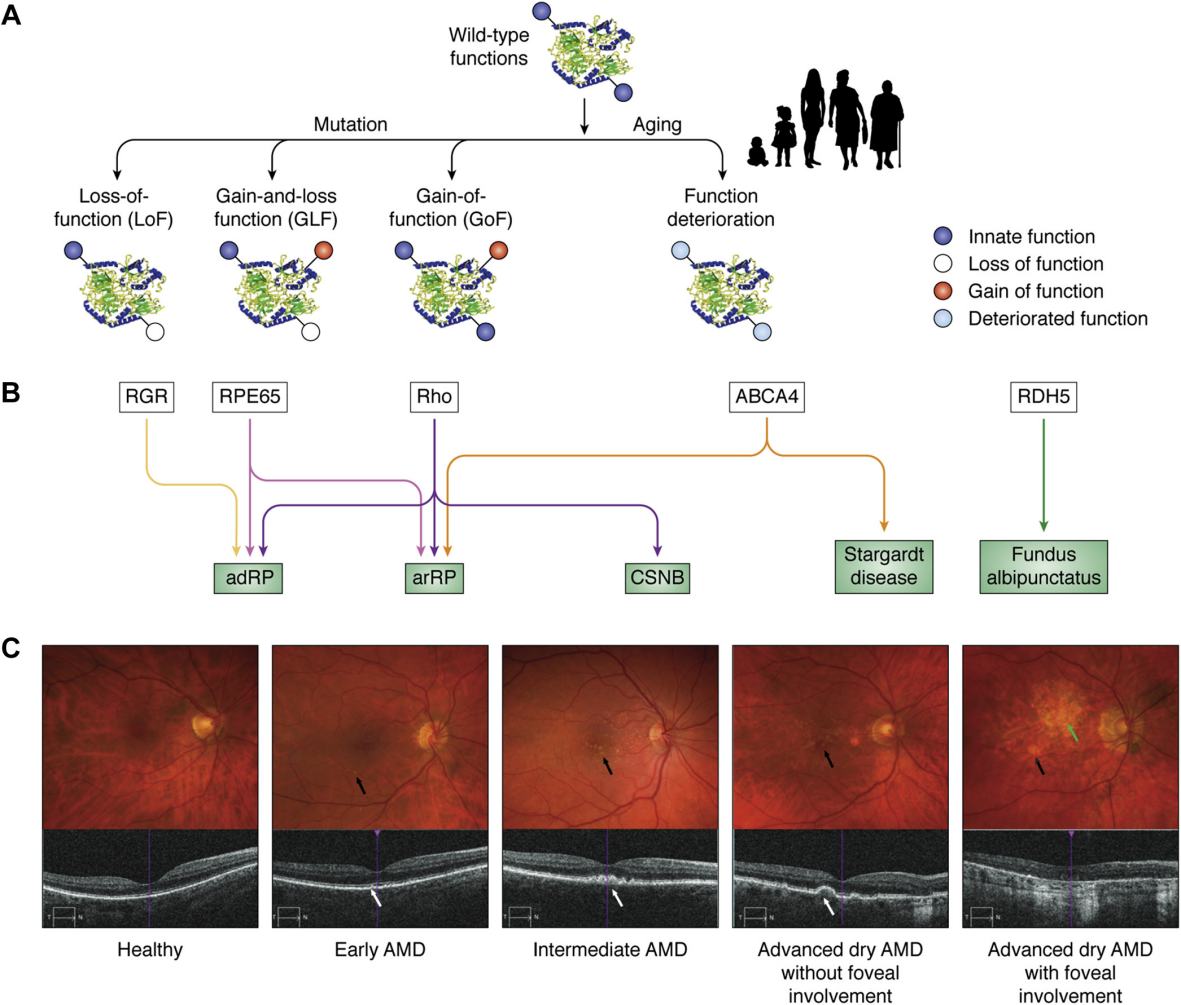
**Genetic disease-causing changes in the visual cycle.***A*, monogenic diseases can be caused by genetic changes such as base substitution, deletion, or insertion. These changes can cause loss of function (LoF) pertinent to normal cellular development and maintenance and visual processing and simultaneous gain and loss of function (GLF) and gain of a new detrimental function (GoF). Aging is a complex process that generally accelerates the deterioration of visual function (*light blue color*) involving many different genes and typically leads to a decrease in chromophore production and concomitant decline in dark adaptation (see (184)). *B*, mutations in one gene can cause several diseases, *e.g.*, mutations in RGR and RPE65 can cause autosomal dominant retinitis pigmentosa (adRP), but a mutation in RPE65 can cause autosomal recessive RP (arRP); changes in rhodopsin (Rho) can lead to adRP, arRP, or congenital stationary night blindness (CSNB); mutations in the ATP-binding cassette subfamily A member 4 (ABCA4) transporter can cause arRP and a unique disease with characteristics of juvenile macular degeneration known as Stargardt disease. Mutations in one gene, *e.g.*, retinol dehydrogenase 5 (RDH5), can cause *Fundus albipunctatus*. Thus, LoF, GLF, and GoF can be associated with one gene that will manifest in different disease states, or one clinically recognized disease can be caused by a mutation in different genes. *C*, age-related macular degeneration (AMD). Alterations in biochemical pathways in the retina, including those comprising the visual cycle, can alter retinal integrity in disease states such as AMD, as shown in the upper row of fundus images and in the lower set of images obtained by optical coherence tomography (OCT). Drusen are not seen beneath the retina in normal eyes. Drusen are biochemical waste products appearing as *yellow spots* on color fundus photographs (*black arrows*) or bumpy elevations in the RPE on OCT (*white arrows*). Drusen are the hallmark of early AMD and increase in size and number in intermediate AMD. In advanced dry AMD, patches of RPE death produce areas of geographic atrophy eccentric to or involving the fovea (*green arrow*). These images are provided to illustrate typical disease features, not to report new research findings.

Functional insights often lag significantly behind initial retinal disease genetic findings because of the complexity of protein activities and the rarity of animal models that fully recapitulate human conditions. Many genes contributing to the visual cycle are associated with inherited retinal diseases such as LCA, rod–cone dystrophy, and a juvenile form of macular degeneration called Stargardt disease (Fig. 3) (90, 91, 92, 93). In the simplest case, the mutation of a gene can lead to a LoF; for example, an inactivating mutation in RDH5 abrogates RDH activity and leads to the disease known as *Fundus albipunctatu*s, characterized by delayed dark adaptation and the appearance of white flecks in the retina (94, 95) (Fig. 3, *A*–*B*).

Many gene products have multiple functions, *e.g.*, rhodopsin (opsin + chromophore) (Rho) is both a light receptor and a structural protein (4, 96). Disabling the chromophore-binding site in opsin (97) results in a constitutively active visual pigment molecule (98), but also changes the structural integrity of the unliganded opsin in the disk membranes (99, 100), thereby leading to a gain-and-loss of function (GLF). Thus, different mutations in one gene can lead to a variety of diseases such as autosomal dominant (adRP) and autosomal recessive retinitis pigmentosa (arRP) and autosomal dominant congenital stationary blindness (CSNB) (101, 102, 103, 104), all of which can be caused by mutations in the opsin gene.

Mutations in the RPE65 gene can cause the loss of its isomerase activity (arRP) (105, 106, 107) or result in deleterious aggregation properties (adRP) (108, 109). Although gene mutations leading to RGR inactivity or deficiency have been associated with rod–cone dystrophy in humans (110), a confounding *cis*-acting mutation in a nearby gene could also underlie the phenotypes observed in these patients (111). However, clinical trials on *RPE65* gene augmentation therapy showed a significant but modest improvement in retinal function (112, 113) and fading effects in patients (114). Mutations in the gene encoding the ATP-binding cassette subfamily A member 4 (ABCA4) transporter are the most frequent mutations associated with Stargardt disease and also can cause some forms of autosomal recessive cone–rod dystrophy (not shown in Fig. 3) (115, 116) and arRP (117) (reviewed in (90, 91, 92, 93, 118, 119)). These examples point to the simple fact that mutations in many genes can result in clinically similar disease phenotypes and that mutations in one gene can manifest in different forms of retinal disease.

In contrast to identified genetic mutations affecting known components of the phototransduction enzymatic cascade and the visual cycle, a more complex picture arises for diseases such as AMD, where aging processes lower the efficiency of visual processing (Fig. 3*A*) (120). The most reliable phenotypic factor, in addition to ocular drusen (Fig. 3*C*), is the delay in dark adaptation that occurs with age (121, 122, 123). Ultimately, routine genetic testing will be more informative than observing the clinical manifestations of the disease alone. However, genetic perturbations as just described can be complex, thus simple mechanistic interpretations may not be possible without a better understanding of the genetics and the array of interacting cellular pathways.

## Retinoid-based treatment of retinal diseases

A number of pharmacologic strategies are currently under development for the treatment of retinal diseases (reviewed in (91, 92, 124)). Here, we briefly review two classes of drugs with opposing effects on visual pigment activity and consequently distinct retinal disease applications.

**Figure 4:**
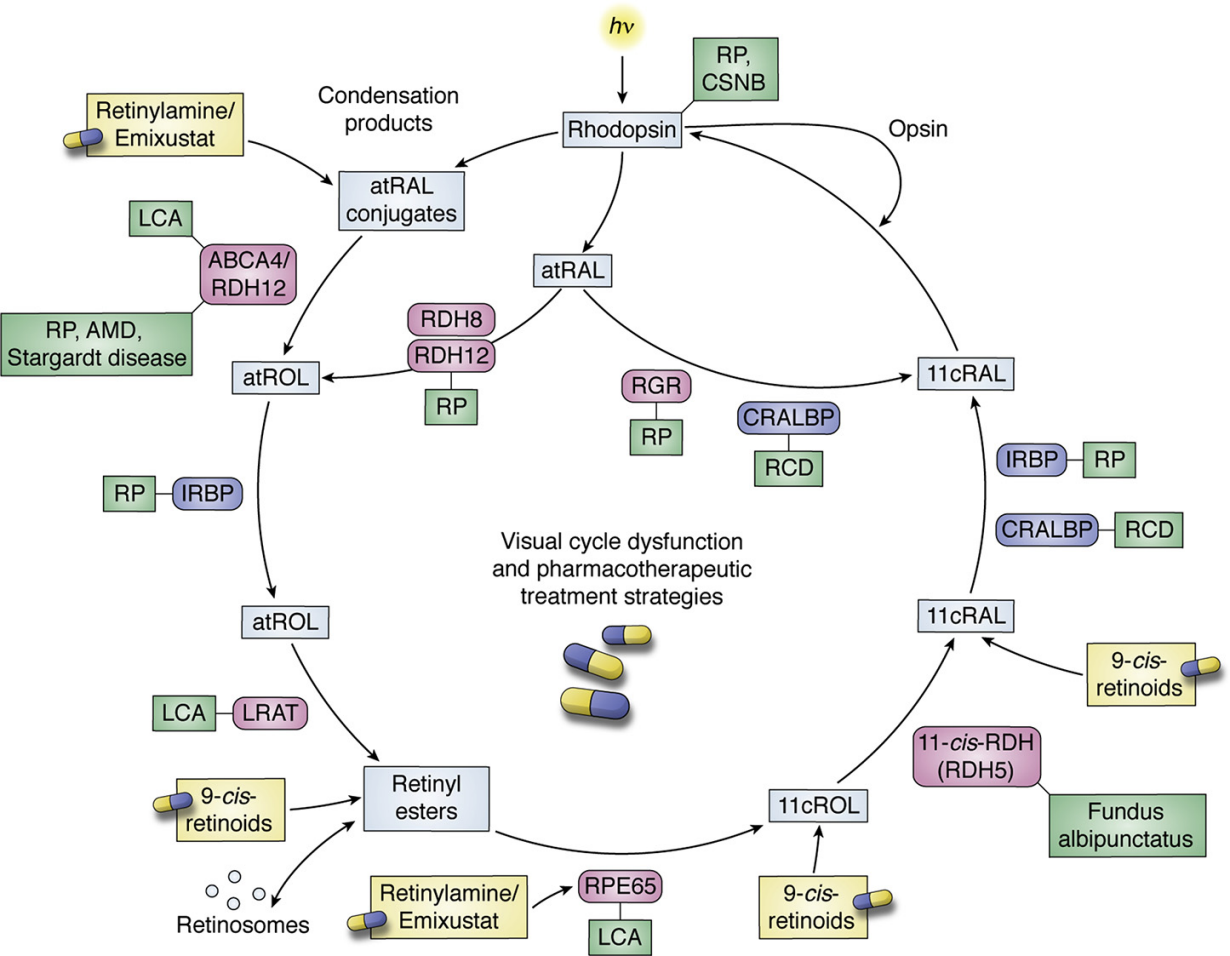
**Diseases involving the visual cycle and pharmacotherapeutic treatment strategies.** Visual cycle dysfunction is associated with a variety of ocular diseases including Leber congenital amaurosis (LCA), retinitis pigmentosa (RP), age-related macular degeneration (AMD), Fundus albipunctatus, rod-cone dystrophy (RCD), cone stationary night blindness (CSNB), and Stargardt disease (shown in *green boxes*). Enzymatic and other protein dysfunctions in the visual cycle can be overcome in some cases by the administration of a 9-*cis*-retinoid (9-*cis*-retinal or 9-*cis*-retinyl acetate), which is an effective visual chromophore substitute. The formation of off-pathway and potentially toxic retinoid adducts (bisretinoids), as occurs in Stargardt disease and AMD, can be slowed by treatment with visual cycle modulators such as emixustat and retinylamine.

The first of these is artificial visual chromophore therapy for the treatment of retinal diseases caused by defects in the visual cycle (Fig. 4). In this approach, exogenous chromophore is supplied to the retina where it can combine with opsin to regenerate visual pigment, thus bypassing the visual cycle metabolic blockade. The 9-*cis*-retinoids, as opposed to the natural chromophore 11-*cis*-retinal, were selected for therapeutic development owing to two main properties: 1) 9-*cis*-retinal readily combines with rod and cone opsins to generate isoRho and iso-cone opsin pigments with spectral properties and quantum yields largely similar to those of native visual pigments (24, 125, 126, 127, 128); and 2) the ease of synthesis (129, 130) and thermal stability of 9-*cis*-retinoid isomers are significantly greater than those of 11-*cis*-retinoids (7). The therapy is typically delivered as a 9-*cis*-retinyl acetate prodrug that undergoes hydrolysis and oxidation *in vivo* to generate the pharmacologically active 9-*cis*-retinal molecule (131) (Fig. 4).

The efficacy of 9-*cis*-retinoid therapy (Fig. 4) for visual-cycle-related diseases has been demonstrated in mouse (132, 133, 134) and dog (10) models of LCA harboring mutations in the RPE65 gene. These findings set the stage for clinical trials in humans with recessive mutations in RPE65 and LRAT where both subjective and objective efficacy measures were demonstrated (11). The most recent application of the therapy has been in the treatment of the Asp^477^Gly RPE65 mutation where it was shown that a 1-week course of 9-*cis*-retinyl acetate could rescue the night blindness associated with this form of adRP for up to 6 months (135). This prolonged duration of action arises from the ability of 9-*cis*-retinoid to be esterified and stably stored in the RPE where it is released over time to continually regenerate visual pigment (131).

On the opposite side of the coin, normal or perhaps overactivity of the visual cycle has been linked to specific retinal diseases including Stargardt disease, diabetic retinopathy, and geographic atrophy. Consequently, there have been significant efforts made to generate inhibitors of the visual cycle for the treatment of these conditions. Owing to its role in the key isomerization reaction as well as the generation of lipofuscin (136, 137), which may drive progression of certain retinopathies, RPE65 has been most heavily targeted for the development of therapeutic visual cycle modulators. The first potent RPE65 inhibitor developed is a molecule called retinylamine, which was designed as a transition-state analog of RPE65 (138). This concept was further refined in the development of the retinylamine derivative known as emixustat (139), which is not a retinoid, but retains key structural elements of the retinylamine backbone (81). Emixustat (Fig. 2 and Movie S2) is approximately ten times more potent than retinylamine *in vitro* (140), is orally bioavailable (141), and exerts specific effects on *in vivo* visual cycle activity as demonstrated in phase 1 and 2 clinical trials (142). It was shown that the potency of emixustat can be further improved by substitutions at the cyclohexyl moiety, for example, in the molecule MB-004 (143). Unfortunately, emixustat failed to demonstrate efficacy in the treatment of geographic atrophy as assessed in a large phase 3 randomized, double-blind, placebo-controlled trial (144). However, this drug continues to be tested in other diseases including Stargardt disease and diabetic retinopathy. Interestingly, retinylamine/emixustat family molecules exhibit a second mechanism of action involving the direct sequestration of retinal released from bleached visual pigments, and this action plays a key role in the ability of these molecules to protect against light-induced retinal degeneration (140, 145). Efforts are currently underway to develop retina-targeted retinaldehyde traps for treatment of diseases associated with formation of toxic retinaldehyde adducts (143, 146).

## Genetic methods to rescue vision

**Figure 5:**
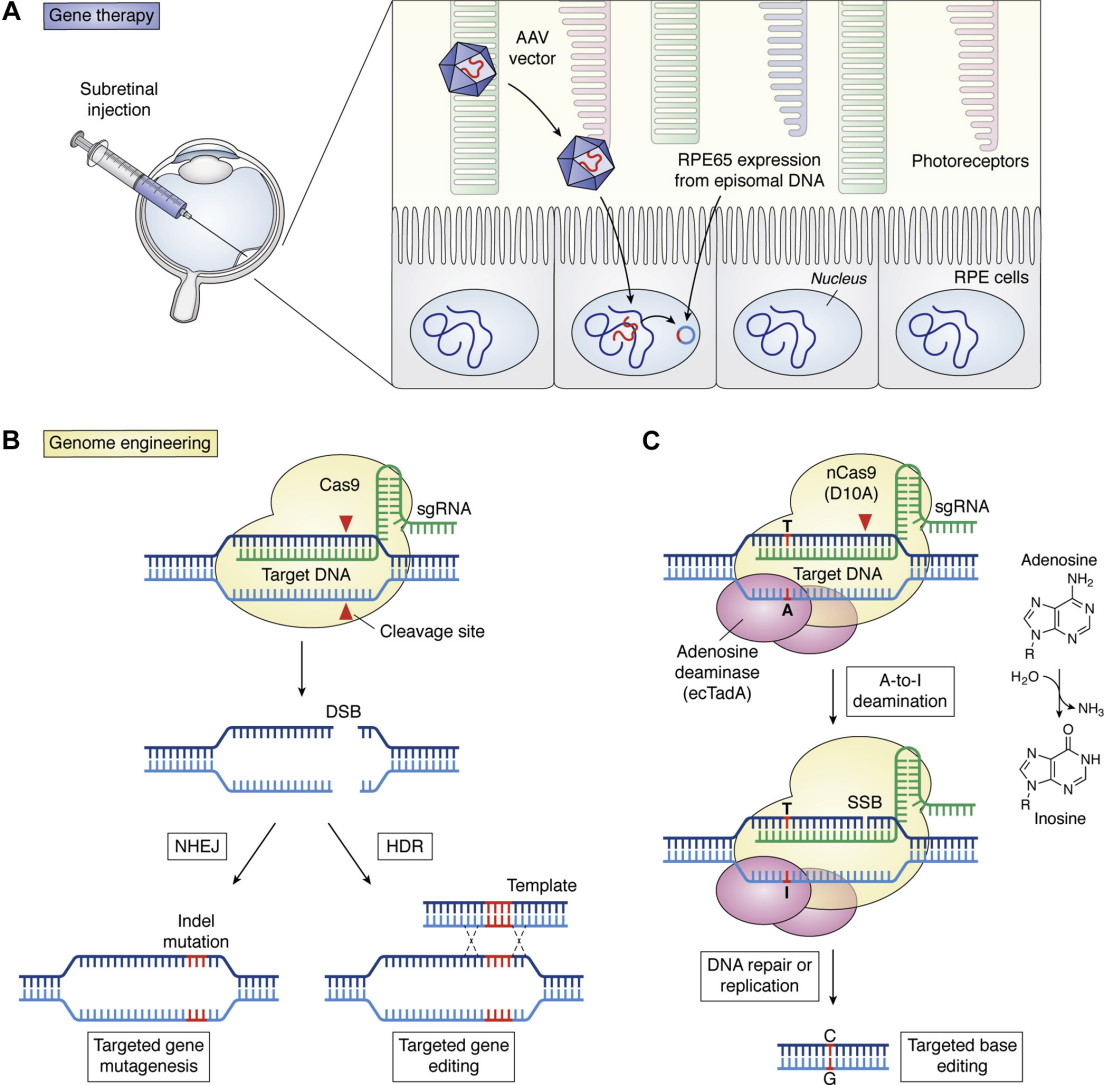
**Pharmacological intervention using genetic methods.***A*, gene augmentation therapy for patients with a defective RPE65 gene (reviewed in (185)). The RPE65 gene is delivered by subretinal injection using an adeno-associated viral (AAV) vector that lacks the capacity for replication. The appropriate tropism of the vector (serotype 2) allows specific transduction of the RPE cells, in which the RPE65 transgene remains episomal to the host DNA. Luxturna (Spark Therapeutics) has been approved as a therapeutic agent to treat Leber congenital amaurosis (LCA) caused by inactivating mutations in RPE65. *B*, genome engineering using the CRISPR-Cas9 system. The bacterial-derived Cas9 nuclease (shaded in *yellow*) complexed with a single-guide RNA (sgRNA, *green*) recognizes and binds to the target DNA that is complementary to the sequence of sgRNA (modified from (186)). Cas9 cleaves double-stranded DNA, which can be repaired in the error-prone nonhomologous end joining (NHEJ) and homology-directed repair (HDR) pathways. However, NHEJ can result in random indel mutations at the site of the junction and can result in frameshifts or a premature stop codon, resulting in gene knockout. In the HDR pathway, the DNA double-stranded breaks can be repaired with a homologous sequence template. *C*, CRISPR-Guided DNA Base Editors. The adenine base editor is constructed in a manner to target DNA by a Cas9 nickase (nCas9), with an adenosine deaminase (ecTadA), which deaminates a target adenosine (A) to inosine (I). Because inosine is recognized as guanosine (G) by the cellular machinery, the adenine base editor leads to conversion of the original A·T base pair to a G·C base pair by a DNA repair process (modified from (187)). Similarly, a cytidine base can be modified to uridine (recognized as thymidine), resulting in a G to A transition.

In an early publication, Ali and colleagues (1996) (147) demonstrated that recombinant particles of low toxicity adeno-associated virus (AAV) encoding β-galactosidase under a cytomegalovirus (CMV) promoter were capable of transducing cells of the RPE and photoreceptors with high yield when injected into the subretinal space. Soon after, the replacement of the CMV promoter with the rod opsin promoter demonstrated 100% photoreceptor transduction at the site of injection, suggesting the feasibility of tissue-specific transduction for gene therapy in the eye (148). At another milestone, gene therapy was employed to rescue rod OS formation in naturally occurring *rds* mice lacking the structural protein peripherin-2 (149). This study demonstrated that genes encoding structural proteins also were amenable to gene therapy. Such technology was successfully extended to a large animal model of a human disease carrying an RPE65 mutation (150) (Fig. 5*A*). Like RPE65, loss of the key visual cycle enzyme LRAT, which is essential for the retention of retinol in the eye, could be overcome by subretinal gene therapy (151). These findings then inspired the successful delivery of genes to patients with blinding diseases that otherwise were inoperable, culminating in the approval by the U.S. Food and Drug Administration of Luxturna (voretigene neparvovec-rzyl), a new gene therapy for patients carrying RPE65-inactivating mutations causing LCA. In this treatment, the viral construct remains in the targeted cells as episomal DNA (Fig. 5*A*). However, clinical trials on *RPE65* gene augmentation therapy showed only a modest improvement in retinal function (112, 113) and fading effects in patients (114). Furthermore, this approach could not prevent further degeneration of photoreceptors despite expression of the *RPE65* gene (152, 153). These studies also required subretinal injections, which could perturb foveal cones during surgery. Intravitreal injection would avoid this complication, and thus efforts to engineer AAV variants were undertaken. Shortly afterward, an AAV variant was identified and then demonstrated to penetrate the retina in retinoschisis and LCA mouse models (154). Similarly, an AAV viral capsid that penetrates the primate retina (155) and could be developed for use in human gene transfer experiments was identified. Several excellent reviews describe other aspects of gene therapy in the eye (12, 156, 157).

There is considerable interest in CRISPR-Cas9 (clustered regularly interspaced short palindromic repeats and CRISPR-associated protein 9)-mediated therapeutic correction of disease-associated mutations (158, 159), so it is not surprising that this approach has been adopted to restore sight in animal models of human LoF diseases (Fig. 5*B*) (160, 161). However, many unresolved issues, including the precision of genome editing and possible off-target effects, remain. For example, DNA double-stranded breaks (DSBs) required for CRISPR-Cas9 technology typically lead to unwanted insertions or deletions at the targeted genomic locus, even if specificity is achieved, mostly resulting in a low yield of correction and heterogeneity of modifications. Future improvements will likely resolve such difficulties.

Examples of CRISPR-Cas9 applications in vision include the ablation of the dominant Ser^334^ter codon Rho mutation while sparing a nonmutated allele in the rat model of adRP (162). In another study, it was postulated that disabling rod genes associated with RP while triggering the expression of cone proteins to salvage both rods and cones could at least partially restore or maintain vision. Neural retina-specific leucine zipper protein (NRL) is responsible for the fate of rods during photoreceptor development (163). Yu *et al.* (164) disrupted NRL using CRISPR-Cas9 in three mouse models of retinal degeneration and observed a significant improvement in rod and cone survival attributable to altered gene expression patterns. CRISPR-Cas9 technology was also employed to remove the aberrant splice donor site in the CEP290 gene that causes a form of LCA (165). Restoration of normal CEP290 expression raises the possibility that this common form of LCA can be rectified by gene editing. In a proof-of-concept study, a mutated *Rpe65* was targeted by CRISPR-Cas9-mediated homology-directed repair (HDR) in a mouse model called *rd12*, which harbors a naturally occurring *Rpe65* mutation (166). The rescue was modest, characterized by approximately 1% homology-directed repair and ∼1.6% deletion of the pathogenic stop codon in the *Rpe65* gene.

A variant of CRISPR-Cas9 technology termed base editing is perhaps even more promising, because in part it relies on enzymatic modification of specific bases in genomic DNA (or RNA) without requiring DSBs (Fig. 5*C*) (167, 168, 169). Base editing is dubbed precision chemistry of the genome. Briefly, a mutant CRISPR targets the genomic DNA without making DSBs. Base editors (cytidine/adenosine deaminase fused to catalytically inactive Cas9) then convert C > T (or A > G) *via* mismatched U:G or I:A pairs, resulting in much lower off-target activity and far fewer alternative mutations compared with traditional Cas9 editing. These deaminases are single-strand-specific, and editing is therefore limited to an editing window near the protospacer adjacent motif (PAM sequence) where the sgRNA–Cas9 complex binds to DNA. Moreover, indel formation is much less frequent when using base editors as compared with traditional Cas9, which induces DSBs (Fig. 5*C*). The targeted mutagenesis using base editors has been highly successful in plants (170, 171, 172, 173, 174, 175), and recently, base editing was utilized to correct a nonsense mutation in the *Rpe65* gene in the *rd12* mouse model with up to 29% efficiency (176). Minimal indel and off-target mutations were observed. The rescue of RPE65 expression, retinoid isomerase activity, and retinal and visual function to near-normal levels in the retina and visual cortex were also demonstrated. This technology could replace simple gene augmentation approaches, at least in some cases, and promises an exciting future for base editing technology.

## Conclusions

Vision research has a rich history of groundbreaking discoveries. Work of George Wald and colleagues on the biochemical underpinnings of visual transduction has led to the most advanced molecular understanding of a sensory system (2, 177). Rho serves as a prototypical GPCR whose mechanism of signaling remains the best studied of any member of the GPCR superfamily (4, 5, 177). This is crucial because GPCRs are the preferred targets for many drugs. Anatomic, genetic, and electrophysiological studies of the retina and visual cortex have led to a greater understanding of the development, organization, function, and plasticity of the nervous system (178, 179). Now, the exquisite molecular details of visual chromophore regeneration are helping to define pharmacological and genetic therapies for blinding diseases and the application of these new approaches to other human disorders and aging. Recent findings demonstrate that cones rely on both continuous RPE65 activity and an independent second source of 11-*cis*-retinal for regeneration of their visual pigments (14). As photic regeneration of 11-*cis*-retinal has been characterized in mollusks and squid, it is conceivable that it could also contribute to chromophore regeneration in vertebrates. RGR was originally reported to mediate a photic visual cycle in vertebrates (180), but low 11-*cis*-retinal synthetic activity cast doubt on its physiological relevance. Now, irrefutable evidence has been obtained that a specific splice variant of RGR exhibits robust isomerization activity comparable with RPE microsomes. RGR does not require additional proteins, rather it can be purified in a form that can be photoactivated and stimulated by CRALBP. Because CRALBP and RGR are expressed in the RPE and Müller cells (68, 181), novel mouse models are needed to dissect the specific role of each cell type during the photic generation of 11-*cis*-retinal (182). Thus, recent progresses in animal models, human genetics, and structural biology have provided essential information about these processes, as well as innovative approaches to treat human blinding disorders. Because of these discoveries, new opportunities now exist for pharmacological treatments (91) and gene therapies (166, 176) to prevent the deterioration of vision or even in some cases the restoration of sight.

## Conflict of interest

The authors declare that they have no conflicts of interest with the contents of this article.

## Supplementary data

Movie S1 Activation of bovine rhodopsin. This movie begins by showing the retinylidene chromophore tightly bound in an interior pocket, sequestered away from the solvent, in ground-state rhodopsin (Protein Data Bank (PDB) accession code: 1U19). Upon light absorption, 11-*cis*-retinal isomerizes to the all-*trans*-configuration resulting in changes to the helical bundle that allows coupling of the receptor to a G protein as shown in the morph (PDB accession code: 3PXO).

Movie S2 Catalytically important features of the RPE65 active site. This movie illustrates features of the active site in RPE65 that are responsible for its regio- and stereoselectivity, including the retinoid-binding site constriction at the C11 position as well as the putative nucleophilic water molecule. The binding position for 11-*cis*-retinol shown in the movie was predicted from computational docking and is in good agreement with the binding positions of the retinoid-mimetic molecules emixustat and MB-001. This movie was used with permission from (81).
